# Novel computational methods for increasing PCR primer design effectiveness in directed sequencing

**DOI:** 10.1186/1471-2105-9-191

**Published:** 2008-04-11

**Authors:** Kelvin Li, Anushka Brownley, Timothy B Stockwell, Karen Beeson, Tina C McIntosh, Dana Busam, Steve Ferriera, Sean Murphy, Samuel Levy

**Affiliations:** 1 The J. Craig Venter Institute, 9704 Medical Center Drive, Rockville, MD 20850, USA

## Abstract

**Background:**

Polymerase chain reaction (PCR) is used in directed sequencing for the discovery of novel polymorphisms. As the first step in PCR directed sequencing, effective PCR primer design is crucial for obtaining high-quality sequence data for target regions. Since current computational primer design tools are not fully tuned with stable underlying laboratory protocols, researchers may still be forced to iteratively optimize protocols for failed amplifications after the primers have been ordered. Furthermore, potentially identifiable factors which contribute to PCR failures have yet to be elucidated. This inefficient approach to primer design is further intensified in a high-throughput laboratory, where hundreds of genes may be targeted in one experiment.

**Results:**

We have developed a fully integrated computational PCR primer design pipeline that plays a key role in our high-throughput directed sequencing pipeline. Investigators may specify target regions defined through a rich set of descriptors, such as Ensembl accessions and arbitrary genomic coordinates. Primer pairs are then selected computationally to produce a minimal amplicon set capable of tiling across the specified target regions. As part of the tiling process, primer pairs are computationally screened to meet the criteria for success with one of two PCR amplification protocols. In the process of improving our sequencing success rate, which currently exceeds 95% for exons, we have discovered novel and accurate computational methods capable of identifying primers that may lead to PCR failures. We reveal the laboratory protocols and their associated, empirically determined computational parameters, as well as describe the novel computational methods which may benefit others in future primer design research.

**Conclusion:**

The high-throughput PCR primer design pipeline has been very successful in providing the basis for high-quality directed sequencing results and for minimizing costs associated with labor and reprocessing. The modular architecture of the primer design software has made it possible to readily integrate additional primer critique tests based on iterative feedback from the laboratory. As a result, the primer design software, coupled with the laboratory protocols, serves as a powerful tool for low and high-throughput primer design to enable successful directed sequencing.

## Background

Directed sequencing is important for the discovery of novel polymorphisms in human DNA sequences that may play a role in disease susceptibility and progression [1]. In order to efficiently screen large numbers of target regions across a diverse panel of DNA samples, a sequencing center needs to apply a high-throughput approach with the goal of minimizing cost. To achieve this, PCR primers are designed with the aim of eliminating human labor associated with amplification protocol optimization and other manual intervention caused by primer failures. Recent contributions in computational PCR primer design include screening additional thermodynamic attributes [2,3] and increasing the genomic annotation [4,5] considered in the design process. Table 1 provides a comparison of novel features of our primer design pipeline in relation to recently published primer design software [3,5-8]. Our primer design pipeline is further enhanced by a higher degree of operational automation and the parameterization of many key attributes in its design. We have employed this pipeline for over five years at our sequencing center and its use was instrumental in the publication of mutations discovered in a set of 20 tyrosine kinase receptor domains consisting of 160 exons, across 19 tumor samples in glioblastomas [9]. Since 2005, over 1.9 Mb of sequence have been targeted with a primer design target coverage rate exceeding 95% (Table 2). Our directed sequencing pipeline has achieved a sequencing success rate of over 90% (Table 3) and we have detected over 7200 potential variations in 150 genes, 3408 of which were not previously recorded in NCBI's dbSNP.

**Table 1:** Comparison of JCVI Primer Design Tool versus other recently published primer design packages.

**Feature/Software**		**Primer Design Assistant (PDA)^3^**	**SNPbox^5^**	**MutScreener^6^**	**EasyExon Primer^7^**	**PrimerZ^8^**	**JCVI Primer Design Tool**
**Year**		2003	2005	2006	2006	2007	2008

**Inputs**	Ensembl ID	-	-	-	-	∗	∗
	DbSNP/AFFY Probe ID	-	-	-	-	dbSNP/AFFY Probe ID	dbSNP ID
	Genomic coordinates	-	-	UCSC annotation	-	-	Ensembl coordinates
	RefSeq ID/Genbank ID	-	Genbank ID	-	Refseq mRNA ID	-	-
	Sequence	∗	∗	∗	-	-	∗
**Primer design parameters validated in lab**		-	-	-	∗	-	∗
**Allows variable depth of coverage**		-	-	-	-	-	∗
**Provides graphical web interface**		∗	∗	∗	∗	∗	-
**Performs dynamic amplicon tiling**		-	‡	‡	‡	‡	∗
**Uses available annotation in primer design**		-	∗	∗	∗	∗	∗
**Checks for stem-loop structures**		∗	-	-	-	-	∗
**Detects phase shifting events in amplicon**		-	stutter only	-	-	-	∗
**Can specify regions to avoid designing primers**		-	-	-	-	-	∗
**Check for alternative amplification**		-	-	-	∗^†^	-	∗
**Enables high-throughput runs**		-	∗	-	-	-	∗

∗ Feature supported

- Feature not supported

^‡ ^Tiling pattern is fixed

^† ^Searches for the full length of the primer, instead of just the critical region on the 3' end

**Table 2:** Size and coverage rates of overall and exonic target regions by project

Projects	# Genes	Total Target Region(bp)	% Target Region Covered	Exonic Target (bp)	% Exon Target Covered
JCVI #1	109	1,609,447	94.71%	380,045	96.39%
JCVI #2	12	203,592	98.39%	65,445	99.55%
JCVI #3	1	14,691	90.27%	847	100.00%
JCVI #4	2	4,724	100.00%	2,723	100.00%
JCVI #5	26	161,631	98.81%	161,631	98.81%

Total	150	1,994,085	95.40%	610,691	97.14%

**Table 3:** Sequencing success rates for projects by amplification protocol and region type

	Standard		High GC		Combined Amplicon Statistics			
			
Projects	# Amp.	% Amp. Success	# Amp.	% Amp. Success	# Total Amp.	% Amp. Success	# Exonic Amp.	% Exonic Amp. Success
			
JCVI #1	3,529	94.87%	1,786	82.42%	5,315	90.69%	1,680	94.52%
JCVI #2	465	93.98%	282	98.94%	747	95.85%	230	96.96%
JCVI #3	12	83.33%	28	100.00%	40	95.00%	10	100.00%
JCVI #4	10	100.00%	10	100.00%	20	100.00%	15	100.00%
JCVI #5	422	95.73%	427	90.16%	849	92.93%	777	96.14%
			
Total	4,438	94.84%	2,533	85.83%	6,971	91.57%	2,712	95.24%

## Implementation

### Computational PCR primer design

#### Software

**Figure 1:**
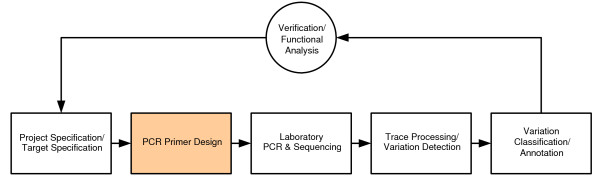
An overview of the JCVI High-throughput Directed Sequencing Pipeline.

In the method described here, we constrain the PCR primer design software to generate a set of primers with a high likelihood of success using one of two standardized amplification protocols. The high-throughput computational PCR primer design pipeline is one of the first computational stages we employ as part of our directed sequencing pipeline (Figure 1).

The computational PCR primer design software is available on the SourceForge.net JCVI Primer Designer website [10]. The tar file of the latest release is available by navigating through the 'Download' tab. It can be installed and run on any computer with a Unix operating system.

#### Inputs

Target regions are specified by the investigator through a variety of identifiers. By using the feature rich Ensembl Perl Application Programming Interface (API) [11], the following types of identifiers are supported as input: Gene, Transcript, HUGO, Exon, and dbSNP. Since the reference genome assembly and annotation may be updated, using identifiers allows us to track features of interest even if their absolute genomic coordinates have changed over time. In addition, by using these identifiers as anchors, we are able to augment target regions with a specified number of flanking bases upstream and downstream of the region, and, if applicable, around exons. The specification of flanking bases, which essentially provides padding around features, implicitly allows the ability to target promoter regions, splice sites, and introns, relative to exons. Since there is also an interest in non-coding regions, we support arbitrary genomic regions as input based on chromosome coordinates. Additionally, the investigator has the option of including evolutionary conserved regions (ECRs) in their target region. We define ECRs as overlapping conserved regions between the human, mouse, and rat genomes established by a set of alignment criteria (>100 bp, >80% sequence identity). The ECRs for the individual species are obtained dynamically from the Dcode ECR Browser website [12,13].

#### Outputs

**Figure 2:**
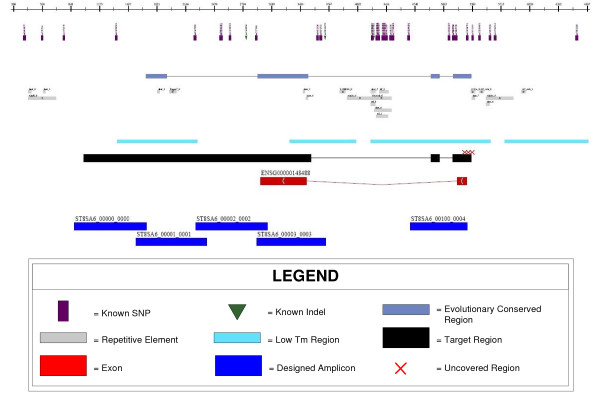
Sample output from a primer design run displaying amplicons in relation to genomic features.

The output of the primer design pipeline is a set of primer pair sequences and their theoretical amplicons based on a reference sequence. In addition, we output a summary of the critique results, such as the primer and amplicon melting temperatures and information about trace phase shifting events. The primers can be optionally 5' tailed with M13, or any other pre-defined, sequencing primers, and are ready for ordering and synthesis. A GFF File [14] is produced to allow the investigator to review the spatial organization of the amplicons in juxtaposition with sequence annotation. The GFF file also highlights target regions where primer pairs could not be designed. This allows the investigator to decide whether to proceed with sequencing, despite having areas that lack coverage, or to initiate a more customized primer design, targeting the uncovered regions. Figure 2 shows an example of a rendered GFF file, in the form of an automatically generated Adobe PDF file, incorporating the Ensembl annotation and designed amplicons. In addition to the visual summary, a statistical overview is also generated which reports the number of amplicons that were designed and their average, minimum, maximum and standard deviation of lengths. For high-throughput primer designs, we have project level tools which summarize the total and percentage of targeted base pairs that were covered.

#### Algorithm

The primer design pipeline is composed of two key software components: the Coverage Manager (CM) and the Primer Critiquor (PC).

The Coverage Manager (CM) is responsible for generating a dynamic amplicon tiling across the supplied target regions. Its parameters include the target, minimum, and maximum amplicon size, the minimum number of overlapping base pairs required between amplicons, and the depth of redundant amplicon coverage required. Overlap between amplicons is typically warranted when using Sanger sequencing because approximately 50 bp from the 5'end of the read may be unusable due to its low quality. As a result, an overlap of at least 100 bp is required between adjacent amplicons to ensure that the target region can be bi-directionally covered with high-quality sequence. The CM employs Primer3 [15] to generate a list of primer pair candidates. The CM will then invoke the Primer Critiquor (PC) to determine whether or not to accept a primer pair candidate. If multiple target regions are proximally located, the CM will subsume the regions with one amplicon. If the target region is smaller than an amplicon, the CM attempts to center that region within the amplicon. Otherwise, the CM will attempt to dynamically generate a tiling from the 5' to 3' end across the target region. After the first amplicon has been selected, every proceeding amplicon will depend on the length and position of the prior upstream amplicon and the required minimum amplicon overlap. In order to assay targeted regions, non-uniform tiling can be justified because of our ability to select primers which will be successful. This greedy method for amplicon tiling is more efficient than a strictly uniformly spaced tiling methodology [5,7,8] because we can maximize coverage while minimizing redundancy. Target regions are left uncovered by amplicons only if all primer pair candidates have been rejected in that region.

The Primer Critiquor (PC) is responsible for determining whether a primer pair passes or fails our selection criteria. The parameters for most of the criteria have been determined based on prior directed sequencing experiments, providing us the advantage of avoiding a "fuzzy" logic based approach to evaluating the potential success of primer pairs [1]. The PC was designed to have each criterion specified in a modular fashion. In the event that a new failure pattern is discovered, a new criterion can easily be incorporated as an additional module. The PC may be invoked iteratively when the CM needs to test primer pairs during a primer design run, or it may be called upon independently to evaluate an arbitrary set of primer pairs generated by methods that are external to the current pipeline.

The following criteria are evaluated by the PC to determine whether to accept a given primer pair:

#### Alternative amplification detection

Alternative amplification occurs during PCR when primers bind to and amplify non-targeted regions. This is especially problematic when using genomic DNA samples. To check for alternative amplification events, we use BLASTN [16], with a word size of 7, to search for the forward and reverse primers on a reference genome. The BLAST alignments are then filtered and accepted as hits if they meet either of the following two criteria: 1) >80% overall identity and a perfect match of 4 bp at the 3' end or 2) a perfect match of 11 bp on the 3' end. The detection of alternative amplification depends on how well characterized the reference genome is or how similar the reference genome is to the target organism.

If the length of a predicted alternative amplicon exceeds a parameterized threshold, its amplification is not considered viable. We determined this threshold to be 1600 bp by comparing post-PCR gel results against the computationally predicted alternative products. We observed multiple instances which indicated that computationally predicted alternative products were false positives when their lengths exceeded this 1600 bp threshold. We consider this to be a conservative threshold because we also observed instances where computationally predicted alternative amplicons shorter than 1600 bp were not observed in the gel. In these cases, the primer binding for the targeted amplicon was more thermodynamically stable and, therefore, out-competed the alternative amplicons. A primer pair is rejected if that pair yields at least one viable alternative amplification product.

Due to the interest in diseases associated with non-coding regions, such as introns, regulatory regions, as well as intergenic regions, it is important to support the ability to target genomic regions rich in repetitive elements. We do not mask repeat regions on the reference genome before searching for primer binding sites, since it is possible for a primer pair to be specific even when one or both of the primers binds to a repetitive region. We employ the following heuristic to expedite alternative amplification detection. If the primer pair can computationally amplify a product in the repeat library, then the primer pair is immediately failed. If either primer in the pair can bind to a repetitive element shorter than 500 bp, then the primer pair is also eliminated. Primers that bind to longer repeats are passed on to the full genome search. This heuristic allows primers to flank short repeats while forcing primer design to tackle long repeats where multiple tiled amplicons are necessary.

#### Trace phase shifting event detection

**Figure 3:**
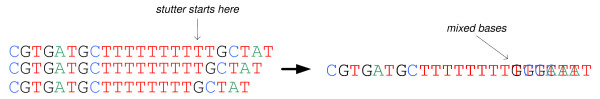
Trace phase shifting events occur when a PCR reaction creates a mixed population of DNA molecules that are then simultaneously sequenced.

A Trace Phase Shifting Event (TPSE) is a DNA sequence motif that may cause Sanger DNA sequencing of PCR amplified products to exhibit mixed peaks 3' of the motif in the chromatogram. This results from a population of amplicons with non-homogenous lengths forming during the amplification process. Two known TPSEs that can be detected result from PCR stutter and insertions/deletions (indels) (Figure 3).

PCR stutter occurs when the polymerase slips during the amplification of mono- or di-nucleotide repeats of a certain length [17]. Instead of the amplification resulting in a uniform population of template amplicon copies, a population of amplicons with mixed lengths, varying at the stutter event site, is generated. We are able to detect sequence potentially prone to slippage by searching for stretches of mono- or di-nucleotide sequence that exceed a specified length threshold. For mono-nucleotide repeats, a stutter event is expected to occur at a length of 8 or greater, N_≥ 8_. For di-nucleotide repeats, stutter events occur when greater than 6 pairs are detected (N^1^N^2^)_≥ 6_, where N^1 ^≠ N^2^.

If the donor DNA sample genotype contains a heterozygous indel in the amplicon, a TPSE will also occur. This results from the amplification process generating a population of two different sequence lengths, for example, one with the indel and one without. We incorporate the indel polymorphism information provided by dbSNP to identify loci that might potentially cause a TPSE.

We have also developed Digital Signal Processing (DSP) software to identify and deconvolve the phase shifted trace files, allowing us to split indels into two synthetic trace files (long vs. short) and to deconvolve the sub-peaks from traces where PCR stutter has occurred (Stockwell, T., manuscript in preparation). As a result of our ability to perform these advanced trace manipulation techniques, the primer design process is permitted to accept a single TPSE per amplicon. Multiple TPSEs are split between separate amplicons if their proximity to each other is greater than the specified required overlap between amplicons. By using this combination of DSP and primer design techniques it is possible to achieve high-quality bi-directional coverage, even in the presence of PCR stutter and indel variants.

**Figure 4:**
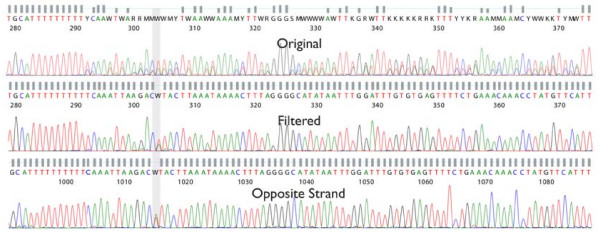
**A trace file with PCR stutter artifact, before (top panel) and after (middle panel) PCR stutter filtering**. The opposite strand trace (bottom panel) is shown in reverse complement, and confirms the discovery of the mixed base.

**Figure 5:**
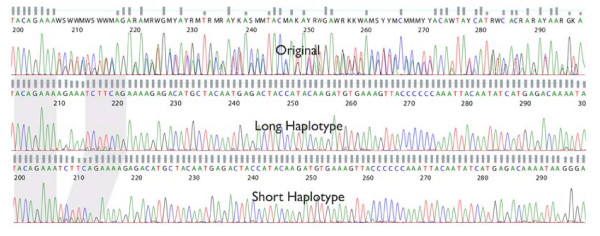
**The trace file in the top panel contains a 5 bp heterozygous indel. The middle and bottom panels show the result of using the correlation-based approach to split the original trace**. The "long" allele trace appears in the middle panel and the "short" allele trace appears in the bottom panel.

Figures 4 and 5 demonstrate the benefits of applying our DSP algorithms to resolving PCR stutter and splitting heterozygous indels. The Figure 4 is an example of how a modified multi-path acoustic echo attenuation filter was used to remove the obfuscated signal resulting from a 10 bp poly-A stutter. The filtered output is able to reveal an A/T variation, that opposite strand sequencing supports, which was indiscernible in the original trace. In Figure 5 is an example of the results of splitting a trace file with a heterozygous indel into its two allelic forms. This was accomplished both by discrete mixed base calling and the de-mixing of the signal using a peak model, as well as by using a correlation-based approach in DNA signal space. An insertion of AGAAA can be detected in the "long" allele, with respect to the "short" allele, after the DSP analysis has been performed.

#### Stem loop interference detection

**Figure 6:**
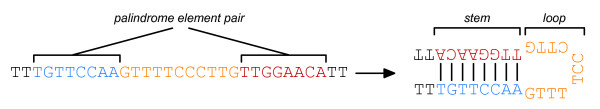
**Identification of a palindrome element pair that could potentially produce stem loop interference**. The stem consists of (3 × 3) + (5 × 2) = 19 hydrogen bonds and the loop has a circumference of 11 bases.

Stem loop interference at the primer binding site is detected with the help of EMBOSS's palindrome algorithm [18]. Thresholds for maximum loop size and minimum stem length were determined based on prior primer pair failures. Empirically derived secondary structure predictions [19] also support the inferred values. If the loop size, as determined by the distance between paired palindrome elements, is less than 11 base pairs and the stem length, as determined by the alignment of the palindrome elements, exceeds 14 hydrogen bonds, then stem loop interference is predicted to occur in the primer binding site (Figure 6).

#### Melting temperature constraints

The sequence melting temperature is computed using the same formulas as those used in Primer3. For primer length sequences, we use the nearest neighbor thermodynamic calculations [20]. To compute the melting temperature of amplicon length sequences, we follow the formula presented by Sambrook et al. [21] which is based on measurements performed by Bolton et al. [22]. We have discovered that, by augmenting the application of the formula slightly, we can improve the ability to further discern potential primer failures. Note that the formula: T_m _= 81.5 + 16.6(log_10_([Na+])) + 0.41 × (%GC) – 600/length, uses a statistical average for the %GC content of the input sequence. The weakness of this averaging is that the amplicon may have localized islands of high GC content that may prohibit complete melting of the amplicon at the predicted T_m_. Our simple modification of this formula's application involves computing the standard deviation of the sequence melting temperature by using a sliding window so that we have multiple %GC samples along the length of the amplicon. This standard deviation is then used in the T_m _formula to estimate the melting temperature variation one standard deviation above the average. If this augmented amplicon melting temperature exceeds our maximum melting temperature cutoff, then the primer pair is rejected.

#### Donor specific variation detection

Donor specific variation in a primer binding site may interfere with primer annealing and decrease PCR efficiency. This can lead to differential success rates across DNA samples or mask mutations as a symptom of allele-specific amplification [23]. Since we are interested in sequencing these polymorphisms instead of inferring donor variation based on PCR success, we design primers to avoid regions of known variability based on information continually updated in dbSNP.

#### Primer dimer detection

Primer dimer is calculated using a dynamic programming algorithm. Although Primer3 performs self and cross primer dimer calculations before generating its list of primer pair candidates, we augment that test by appending the sequencing primer, if necessary, onto the PCR primer before computing primer dimer. This detects internal primer dimers that could have been introduced by the tailing of the PCR primers with the sequencing primers.

### PCR amplification and sequencing protocol

Primer design is performed with the expectation that only two protocols will be required to sequence the majority of target regions. The "Standard" protocol best amplifies regions with a percent GC content centered around 50%. For regions with higher melting temperatures, the "High GC" protocol is used. Depending on the target regions, the ratio of amplicons that are designed for each protocol may vary. Target regions are computationally split into two sets of regions, one for each protocol. Primer design is then executed on each set of regions with parameters calibrated for each protocol. The "Standard" and "High GC" protocols produce amplicons with optimal lengths of 750 bp and 350 bp, respectively. We generate amplicons shorter than the length of a sequencing read in order to achieve full bi-directional coverage. This coverage redundancy increases the accuracy of downstream computational variation detection, therefore, decreasing the amount of manual review labor necessary to the curate indeterminate base calls. This reduction of labor costs more than offsets the additional costs associated with bi-directional sequencing.

"Standard" GC PCR was set up in 10 μL reactions containing each forward and reverse PCR primer at 0.06 μM (Sigma-Genosys, The Woodlands, TX), 1× GeneAmp^® ^PCR Gold Buffer (Applied Biosystems), 2 mM MgCl_2 _(Applied Biosystems), 0.25 units AmpliTaq^® ^Gold DNA Polymerase (Applied Biosystems), 0.2 mM mixed dNTPs (Applied Biosystems), 8% glycerol (Sigma-Aldrich), molecular biology grade water (Quality Biological) and 5 ng of genomic DNA. "High GC" regions were amplified in a 10 μL reaction containing each forward and reverse PCR primer at 0.18 μM, 1× GeneAmp^® ^PCR Gold Buffer, 2 mM MgCl_2_, 0.375 units of AmpliTaq^® ^Gold DNA Polymerase, 0.2 mM mixed dNTPs, 8% glycerol, molecular biology grade water and 30 ng of genomic DNA. Amplification was done in 384-well format on dual 384-well GeneAmp^® ^PCR System 9700 thermal cyclers (Applied Biosystems) with the following cycling conditions: 1 cycle of 96°C (5 min); 40 cycles of 94°C (30 sec), 60°C (45 sec), and 72°C (45 sec) and 1 cycle of 72°C (10 min). After amplification, unincorporated dNTPs were dephosphorylated and excess primers were removed from "Standard" products using a SAP/Exo I reaction containing 0.5 units of shrimp alkaline phosphatase (USB Corporation), 1.0 unit of exonuclease I (USB Corporation) and molecular biology grade water. "High GC" products were treated with 0.5 units of shrimp alkaline phosphatase, 1.76 units of exonuclease I and molecular biology grade water. The SAP/Exo clean up was carried out in a total volume of 15 μL, in 384-well plate format. The cycling program for "Standard" products was: 1 cycle of 37°C (45 min) and 1 cycle of 47°C (15 min) and 72°C (15 min); for "High GC" products it was: 1 cycle of 37°C (45 min) and 1 cycle of 50°C (15 min) and 72°C (15 min).

All PCR products were sequenced in 3.5 μL reactions containing 0.46 μM M13 sequencing primer (Operon Biotechnologies), 0.71× sequencing buffer with sucrose, molecular biology grade water and 0.125 μL of BigDye^® ^Terminator v3.1 Ready Reaction Mix (Applied Biosystems). Sequencing reactions were carried out in 384-well format and thermal cycled using dual 384-well GeneAmp^® ^PCR System 9700s using the following sequencing program: 1 cycle of 96°C (2 min) and 35 cycles of 96°C (10 sec), 50°C (30 sec), and 60°C (1 min). Reaction products were precipitated with a sodium acetate/ethanol mixture followed by a final ethanol rinse and dried at room temperature.

Biomek FX robots (Beckman-Coulter) and Pixsys 4200 nanoliter liquid handling systems (Cartesian Technologies) were employed in setting up PCR, PCR clean up, sequencing reactions and precipitation.

Resuspended sequencing reactions were sequenced on 3730*xl *DNA Analyzers (Applied Biosystems). Immediately before sequencing, the precipitated sequencing reactions were resuspended in 10 μL of 0.75 mM EDTA (Sigma-Aldrich) using a μFill™ (Bio-Tek^® ^Instruments) and shaken using a Microplate Shaker (Union Scientific™ Corporation).

We used either DNA samples supplied by the investigators or obtained from the Coriell Institute to sequence the target regions for all projects. Coriell samples were acquired from the Coriell Cell Repositories [24] and were employed as a readily accessible source of anonymous DNA samples approved for biomedical research. We have also used Coriell DNA samples for both positive controls and for the preliminary testing of primers across an ethnically diverse DNA panel. Since primers are selected based on a reference genome, preliminary testing is useful to receive feedback on the possibility of unexpected primer failures due to variations yet undocumented in dbSNP.

## Results

The primer design pipeline was used to generate primer sets targeting 1,994,085 bp of human genome sequence across 150 genes in 5 separate JCVI projects. JCVI projects #1 and #2 consisted of 121 genes where, for each gene, the regions targeted for primer design consisted of exons, intronic ECRs and 2 kbp upstream and downstream of the first and last exon, respectively. JCVI project #3 targeted one gene in full and included ~2.5 kbp upstream of the first exon and 500 bp downstream of the last exon. For JCVI project #4, which consisted of two genes, primers were designed to target only the promoter region of one gene and only the coding region of the other gene. Primers pairs for the last project, JCVI #5, were designed to target only the exons and splice sites of 26 genes.

**Figure 7:**
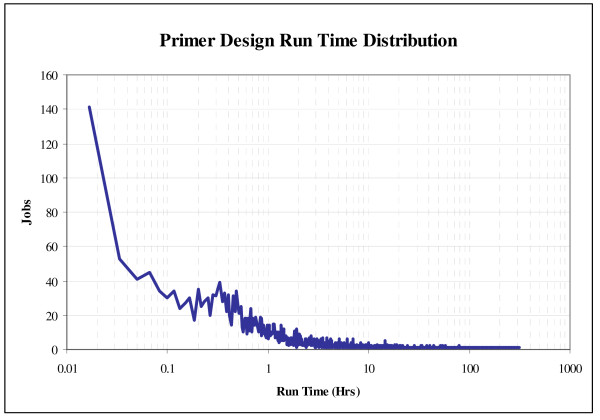
**Primer design run time distribution**. Note that most jobs complete within the first hour.

Primer design is a massively parallel process. Since noncontiguous target regions are independent, primer pairs for each individual target region can be designed on separate processors. In 2006 and 2007, our production pipeline generated 3,385 independent jobs, designing primer pairs across 2,556,071 bp of target region. We utilized 95 compute nodes, each of which were comprised of four 3.06 GHz Intel^® ^Xeon™ processors with 512 kB L2 caches and 2 GB of shared physical memory. Overall, 42% of the jobs completed in less than 1 hour, with a median run time of 1.5 hours (Figure 7). Because of the variability of repeat density and/or other genomic characteristics that tend to increase run time, we observed a positive skew. Any job exceeding 240 hours was typically terminated. For the analysis presented here, only 8 jobs were halted after exceeding this limit. Jobs that require these longer run times are typically a result of target regions densely flanked with repetitive sequence. Since the tiling algorithm will not abandon a portion of the target region until a successful candidate primer pair has been found or until all candidates have been assessed, a region that is impossible to select successful primers for may need to undergo the slow primer specificity search for thousands of unsuccessful primer pairs.

## Discussion

We have developed an automated PCR primer design pipeline that incorporates novel computational methods for improving the prediction of primer pair sequencing success. These computational methods have been parameterized and finely tuned based on the results of our high-throughput directed sequencing experiments, using genomic DNA with Sanger sequencing. The fine tuning of primer pair selection has lead to our high sequencing success rate and our ability to hone our pipeline for the optimal coverage of targeted regions.

The success rate of any primer design pipeline should not only include the amplification success rate of the designed primers, but should also take into account the target regions that could have been covered, but were missed due to overly stringent design criteria. Since we have laboratory data to support the parameters of primer design, we were able to more precisely predict potential amplification errors, thus improving our success rate without unnecessarily compromising target region coverage. It is commonly observed that, as the density of repetitive elements and low complexity sequence increase in intronic and intergenic regions, the ability to design successful primers decreases, therefore reducing coverage. As a result, it tends to be easier to design primers for exonic regions.

Our ability to visualize the designed amplicons aligned with sequence annotation helps to attribute uncovered regions to genomic landmarks, such as repeats (Figure 2). This can minimize the concern of leaving regions uncovered. Our primer design pipeline has achieved an overall target region coverage rate of 95% (Table 2). The exonic subset of this target region has a coverage rate of 97% (Table 2). Compared to existing publications detailing exon coverage results, our corresponding coverage rate is on par with the best of other primer design software [5].

The success rate of designed primers will also vary depending on the chosen criteria for success. For the results presented, we consider a primer pair successful if the expected sequencing result is generated from both sequencing directions by at least two donors in a given a DNA panel. This criterion for success is more stringent than those used by other similar tools during primer validation [5]. In Table 3, we present the results of the recent large scale studies performed on 150 genes. Previously published primer pair success rates focused primarily on the directed sequencing of exons. In order to make our results more comparable, we also calculated our success rate for amplicons associated with exons of the genes in our large scale study (Table 3). We consider an amplicon to be exonic if more than half of the amplicon overlaps with an exon or at least one exon is fully contained within the amplicon. Even with this strict criterion for success, we still achieve an overall exonic sequencing success rate of greater than 95%, which is comparable to the gel-only quantified success rate in [5], or an improvement over other [25] primer design studies.

The modular and parameterized implementation of the primer design pipeline has not only made the computational parallelization of the code possible, but has also allowed us to apply its componentized functionality to other projects. In addition to targeted Sanger sequencing on human subjects, we have designed successful primer pairs in the genome sequences of mouse, dog and *Staphylococcus aureus*. We have also successfully designed primer pairs, with this pipeline, that were subsequently employed in 454 pyrosequencing.

### Future work

The step that requires the largest amount of compute time is the alternative amplification detection, which is done with BLASTN. Our ongoing research suggests that our primer binding criteria may be more stringent than necessary. Currently, our criteria allows for mismatches within the first 8 bases of the primers' 3' end, which requires a more computationally expensive algorithm than perfect string matching. However, crystal structures of Taq DNA polymerase I in a binary complex with primer and template DNA [26] may indicate that the polymerase only needs to interact with the first 8 bases on the 3' end of the primer. Stronger base pairing on the 5' end of the primer will increase the thermodynamic stability of the primer-template complex, leading to more efficient priming and a target amplicon which can out-compete alternative amplicons. However, it is not required to initiate the DNA synthesis that can also be responsible for non-specific amplification.

We have, however, recorded instances of alternative products that resulted despite mismatches within the first 8 bases of the primer's 3' end. Analysis of these alternative products revealed that they did not occur because of nonspecific binding of these primers, but because the sequence of the target amplicon could be found elsewhere on the genome with a percent identity greater than 96%. We hypothesize that a "megaprimer" [27] could be responsible for amplifying the non-target sequence. Megapriming occurs when the DNA copy strand is not completely synthesized before melting from the target template. The partial copy acts as a primer in the subsequent annealing step of the PCR cycle and if the megaprimer anneals to a homologous position on the genome, then this will result in the amplification of the alternative product.

Additional experiments will be performed to determine whether the computational detection of megapriming coupled with an exact match primer binding search will improve the compute time of the primer specificity check while maintaining our high degree of primer specificity.

## Conclusion

We have developed a high-throughput PCR primer design pipeline providing high-quality directed sequencing results while minimizing costs associated with labor and reprocessing. The consistent performance of our laboratory protocols has made it possible to isolate and analyze failures, and to systematically increase our exonic sequencing success rate to 95%. We can readily incorporate new primer critique tests based on iterative feedback from the laboratory, and integrate with downstream processes as a result of the modular software design. This work bridges the gap between computational theory and laboratory practice, and discloses the novel set of computational methods that made it possible.

## Availability and requirements

**Project name: **JCVI Primer Designer

**Project home page: **

**Operating system**: Tested and in production on Linux.

**Programming language: **Perl

**License: **GNU GPL

**Any restrictions to use by non-academics: **None

## Authors' contributions

SM, SL, and KL conceived of the study and participated in its design. KL wrote the software. AB, KL, and TS analyzed data and evaluated results. KB, TCM, DB, and SF designed laboratory protocols, ran experiments, and generated data. KL and AB wrote the manuscript. All authors read and approved the submitted manuscript.
